# Supplementary material for: Possible Applications for a Biodegradable Magnesium Membrane in Alveolar Ridge Augmentation–Retrospective Case Report with Two Years of Follow-Up
Source: Medicina (Kaunas). 2023 Sep 22;59(10):1698. doi: 10.3390/medicina59101698 (PMC10608771; doi:10.3390/medicina59101698)
Supplement: Supplementary file 1 [file medicina-59-01698-s001.zip › medicina-2559681-supplementary/Supplementary Tables.pdf]

Supplementary Tables

Supplementary Table S1: Radiographic evaluation of Case #1		
	Baseline	six-month gain
Volume (cm^3)	na	0.12
Vertical[1] (mm)	na	0.79
Horizontal 1[2] (mm)	2.05	0.93
Horizontal 2[3] (mm)	3.24	1.23
Horizontal 3[4] (mm)	3.67	1.38

[1]measured midcrestally parallel to the long axis of the edentulous ridge, [2]measured perpendicularly to the long axis of the edentulous ridge 1 mm apical to the alveolar crest, [3]measured perpendicularly to the long axis of the edentulous ridge 2 mm apical to the alveolar crest, [4]measured perpendicularly to the long axis of the edentulous ridge 3 mm apical to the alveolar crest

Supplementary Table S2: Radiographic evaluation of Case #2		
	Baseline	six-month gain
Volume (cm^3)	na	0.36
Vertical[1] (mm)	na	2.77
Horizontal 1[2] (mm)	1.74	4.37
Horizontal 2[3] (mm)	3.34	4.33
Horizontal 3[4] (mm)	5.28	3.37

[1]measured midcrestally parallel to the long axis of the edentulous ridge, [2]measured perpendicularly to the long axis of the edentulous ridge 1 mm apical to the alveolar crest, [3]measured perpendicularly to the long axis of the edentulous ridge 2 mm apical to the alveolar crest, [4]measured perpendicularly to the long axis of the edentulous ridge 3 mm apical to the alveolar crest
