# Supplementary figures and images for: Possible Applications for a Biodegradable Magnesium Membrane in Alveolar Ridge Augmentation–Retrospective Case Report with Two Years of Follow-Up
Source: Medicina (Kaunas). 2023 Sep 22;59(10):1698. doi: 10.3390/medicina59101698 (PMC10608771; doi:10.3390/medicina59101698)

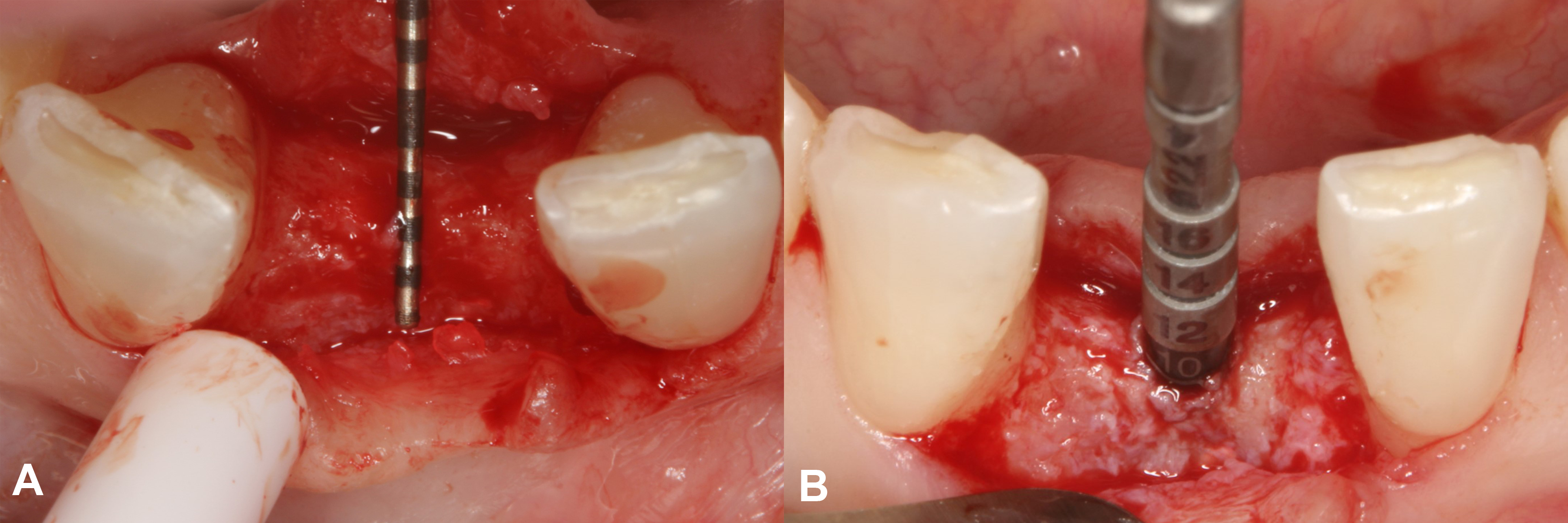

Supplement: Supplementary file 1 [file medicina-59-01698-s001.zip › medicina-2559681-supplementary/Figure S1.tif]

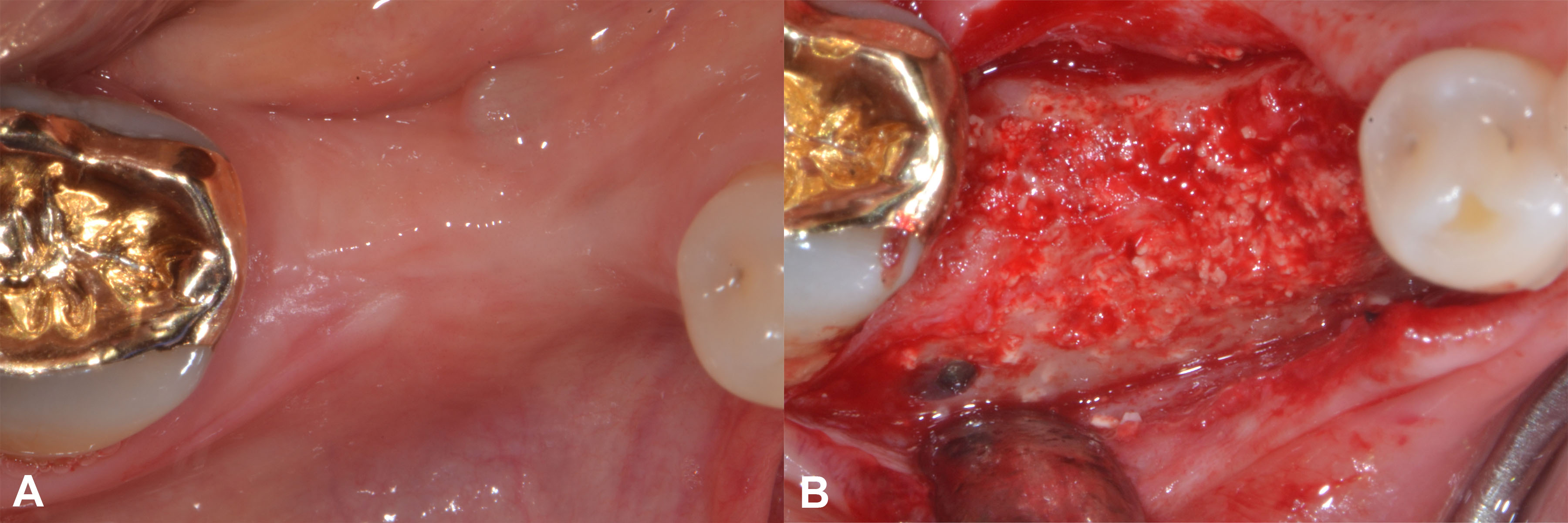

Supplement: Supplementary file 1 [file medicina-59-01698-s001.zip › medicina-2559681-supplementary/Figure S2.tif]
